# Dental Law

**Published:** 1891-05

**Authors:** 


### Dental Law.

THEY are trying to pass a new and better dental law through the Illinois Legislature, and a Chicago dentist is making an unenviable reputation by opposing in every way he can the passage of the bill, on the ground that it makes the requirements so rigid that no one but college educated men, will be able to enter the profession hereafter. And because the course of study in the colleges has recently been extended so as to require three years of study for graduation, no poor boys will be able hereafter to take this college course, because of the expense, and therefore no poor boys will get into the profession. On this basis he stigmatizes this bill under the specious argument that it is class legislation. Of course, we all know that such arguments ought not to influence legislators, but we fear they will. If the poor boy wants to get into the profession he will be willing to work and pay for it too, if he knows there is no other way of honorable entrance. And the same will be true of the boy that can spare the time and money without inconvenience. There is no poor boy that is in earnest and willing to do his best, but will find some sympathizing friends to help him if he will prove himself worthy. It would be a shame and a disgrace to the profession to lower the standard of qualification on any such grounds. The higher the standard of attainment required for permission to practice, the more reliable will be the service to the general public, the most interested party. There is also another phase of the question that the profession is deeply interested in; namely, the dignity and general repute of the profession itself among other professions and the general public. We owe it to ourselves that our calling shall take no mean rank as to scientific attainment; if we are content to class ourselves with the trades, we should give up the professional idea, and quit conferring degrees, that does or should be, significant of something more than mere handicraft. Make the qualification high enough to keep out all badly qualified men and to stimulate bright, intelligent boys to do their best to enter a profession which will honor them in proportion to the rank they attain in it.—H.

